# Development and Characterization of Treprostinil Palmitil Inhalation Aerosol for the Investigational Treatment of Pulmonary Arterial Hypertension

**DOI:** 10.3390/ijms22020548

**Published:** 2021-01-07

**Authors:** Adam J. Plaunt, Sadikul Islam, Tony Macaluso, Helena Gauani, Thomas Baker, Donald Chun, Veronica Viramontes, Christina Chang, Michel R. Corboz, Richard W. Chapman, Zhili Li, David C. Cipolla, Walter R. Perkins, Vladimir S. Malinin

**Affiliations:** Insmed Incorporated, Bridgewater, NJ 08807, USA; adam.plaunt@insmed.com (A.J.P.); Sadikul.Islam@Insmed.com (S.I.); Antonio.Macaluso@Insmed.com (T.M.); Helena.Gauani@insmed.com (H.G.); Tomas.Baker@insmed.com (T.B.); Donald.Chun@insmed.com (D.C.); Veronica.Viranontes@insmed.com (V.V.); Christina.Chang@Insmed.com (C.C.); Michel.Corboz@Insmed.com (M.R.C.); Richard.Chapman@Insmed.com (R.W.C.); Zhili.Li@insmed.com (Z.L.); David.Cipolla@Insmed.com (D.C.C.); Walter.Perkins@Insmed.com (W.R.P.)

**Keywords:** treprostinil palmitil, metered-dose inhaler, prodrug, inhalation aerosol, pulmonary hypertension, pulmonary arterial hypertension

## Abstract

Treprostinil palmitil (TP) is a prodrug of treprostinil (TRE), a pulmonary vasodilator that has been previously formulated for inhaled administration via a nebulizer. TP demonstrates a sustained presence in the lungs with reduced systemic exposure and prolonged inhibition of hypoxia-induced pulmonary vasoconstriction in vivo. Here, we report on re-formulation efforts to develop a more convenient solution-based metered-dose inhaler (MDI) formulation of TP, a treprostinil palmitil inhalation aerosol (TPIA) that matches the pharmacokinetic (PK) and efficacy profile of a nebulized TP formulation, treprostinil palmitil inhalation suspension (TPIS). MDI canisters were manufactured using a two-stage filling method. Aerosol performance, formulation solubility, and chemical stability assays were utilized for in vitro evaluation. For in vivo studies, TPIA formulations were delivered to rodents using an inhalation tower modified for MDI delivery. Using an iterative process involving evaluation of formulation performance in vitro (TP and excipient solubility, chemical stability, physical stability, and aerosol properties) and confirmatory testing in vivo (rat PK and efficacy, guinea pig cough), a promising formulation was identified. The optimized formulation, TPIA-W, demonstrates uniform in vitro drug delivery, a PK profile suitable for a once-daily administration, efficacy lasting at least 12 h in a hypoxic challenge model, and a significantly higher cough threshold than the parent drug treprostinil.

## 1. Introduction

Treprostinil (TRE), a prostacyclin pulmonary vasodilator, has been approved for the treatment of pulmonary arterial hypertension (PAH) [1,2] and is available as a solution for inhalation (Tyvaso^®^, United Therapeutics) [3], as an oral tablet (Orenitram^®^, United Therapeutics) [4], and as an injection (Remodulin^®^, United Therapeutics) [5]. However, because of its short elimination half-life (≈ 30 min), inhalation treatment of PAH with TRE requires frequent dosing to sustain pulmonary vasodilation [3,5]. In addition, inhalation of TRE is associated with adverse local and systemic events, including cough, headache, and throat irritation [1]. 

To overcome these shortcomings researchers, have evaluated numerous strategies to develop alternative therapies, including prodrug chemical modification of TRE [6,7], liposomal encapsulation [8,9], new devices [10], and particle engineering via modern manufacturing techniques [11,12]. Furthermore, to increase convenience and improve patient compliance, there have been preclinical efforts to develop inhaled TRE therapies that utilize pressurized metered-dose inhalers [13] or dry powder inhalers [14]. We hypothesized that a prodrug formulation of TRE delivered by inhalation and designed to provide prolonged efficacy with potentially reduced adverse events following a simple administration procedure could provide a superior treatment option for PAH patients.

**Figure 1:**
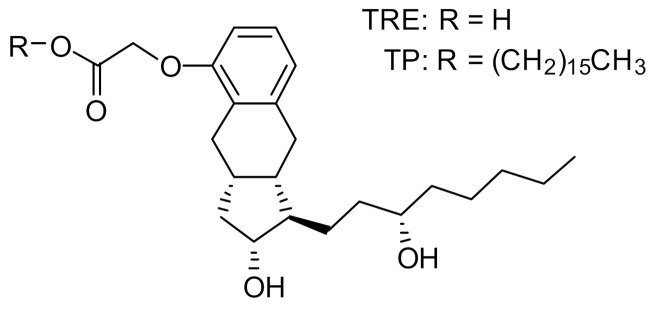
Chemical structure of treprostinil (TRE) and treprostinil palmitil (TP).

Insmed has developed treprostinil palmitil (TP), an ester prodrug of TRE, which hydrolyzes slowly to provide sustained release of TRE over an extended period [15]. The chemical structures of TP and TRE are shown in Figure 1. The initial formulation efforts were focused on the development of a solid lipid nanoparticle that would be delivered directly to the lungs via nebulization [15,16]. The nebulized formulation, referred to as treprostinil palmitil inhalation suspension (TPIS), exhibited a prolonged efficacious response inhibiting pulmonary vasoconstriction in rodents for up to 24 h after administration [16,17] and reduced tachyphylaxis relative to inhaled TRE [16,18,19]. Furthermore, the vasodilatory effect following pulmonary administration of TPIS was associated with significantly lower systemic TRE levels as compared to intravenous TRE administration [17]. This suggests that the magnitude of pulmonary vasodilation is dictated not by systemic circulation of TRE, but rather by the concentration of TRE and TP in the lungs. Notably, this formulation showed promise through Phase I clinical trials [18,19].

While nebulization can be an effective strategy to deliver drugs to the lungs as aerosols, it requires the use of a power-driven device, which must be cleaned daily [20]. To potentially offer a more convenient route of administration, we evaluated the use of pressurized metered-dose inhalers (MDIs) for pulmonary delivery of TP. We hypothesized that a simple solution-based MDI formulation comprised of the TP prodrug, an alcohol cosolvent, and a minimal number of excipients could effectively provide long-lasting vasodilation with reduced adverse events. As part of our development strategy, we used an iterative process involving evaluation of formulation performance in vitro (TP and excipient solubility, chemical stability, physical stability, and aerosol properties) to identify formulations that were suitable for confirmatory in vivo studies (rat pharmacokinetics (PK) and efficacy, guinea pig cough). Key criteria to measure success were defined as follows: (1) slow clearance of TP from the lungs to sustain efficacious lung TRE levels for up to 24 h, (2) negligible progressive accumulation of TP in the lungs, and (3) plasma TRE C_max_ values significantly lower than TRE delivered by the MDI.

## 2. Results

### 2.1. Formulation Development

Using an iterative process involving evaluation of in vitro formulation performance (solubility, stability, and aerosol properties) and confirmatory in vivo studies (rat PK and efficacy, guinea pig cough), we were able to demonstrate chemical stability, physical stability, aerosol performance, PK, and efficacy characteristics. The lead formulation, TPIA-W, is composed of TP, DSPE-PEG2000, PEG400, isopropyl alcohol (IPA), and hydrofluoroalkane HFA-134a. A variety of challenges, including chemical stability (degradation of Active Pharmaceutical Ingredient (API) and excipients), physical stability (i.e., solubility), and poor aerosol performance, were overcome during development. Furthermore, we have shown that the presence of two novel MDI excipients, DSPE-PEG2000 and PEG400, is critical for obtaining the desired in vivo performance of an MDI formulation of TP.

In selecting an appropriate formulation, our goal was to ensure complete dissolution under normal storage conditions and when subjected to cooling–heating cycles. Since TP has limited solubility in pure commercially available hydrofluoroalkane (HFA) propellants (HFA-134a, HFA-227ea, or HP-152a), the use of an alcohol cosolvent was required. The exact amount of alcohol cosolvent needed to ensure a stable solution state in an MDI formulation varied based on the presence of other excipients. We used two types of excipients: Excipient 1 was a non-ionic surfactant (pegylated lipid), and Excipient 2 was a hydrophilic glycol (polyethylene glycol (PEG) or propylene glycol (PG)). A list of evaluated formulations is shown in Table 1.

As expected, the solubility profile of each specific formulation was dependent on a number of factors, including the relative concentration of all formulation components, the concentration of alcohol cosolvent, and temperature. In the absence of excipient, as much as 13% *w/w* ethanol (EtOH) was required to keep TP soluble in HFA-134a. However, when DSPE-PEG2000 and PEG400 were introduced into the formulation, the amount of alcohol cosolvent required could be reduced to 10% while maintaining acceptable physical stability (formulations TPIA-E and TPIA-W). Most formulations tested were soluble at ambient temperature for a period of time after canister filling, but failed to remain soluble (i.e., displayed poor physical stability) during longer-term storage (>1 week).

We observed the formation of a heterogenous suspension for each formulation tested at low enough temperatures, typically –20 °C. Unstable formulations typically demonstrated a precipitation ring along the liquid–vapor interface during storage, though free-floating insoluble material was also occasionally observed. Consequently, our focus shifted towards ensuring re-dissolution of precipitated materials after a short thermal equilibration period. We designed an experiment where canisters were equilibrated for 24 h at 5 °C and then recorded the length of time required for the canisters to present as homogeneous solutions. In the case of TPIA-W, it took less than 30 min exposure to a room-temperature setting for the formulation to present as a homogenous solution. We also designed the converse experiment, where canisters equilibrated at room temperature were stored under refrigerated conditions, and the amount of time to induce precipitation was recorded. In the case of TPIA-W, it took approximately four hours for precipitation to occur (Supplementary Table S1). During thermal cycling studies, TPIA-W consistently presented as a heterogeneous suspension at reduced temperature (–20 °C), but as a homogeneous solution at elevated temperature (40 °C).

**Figure 2:**
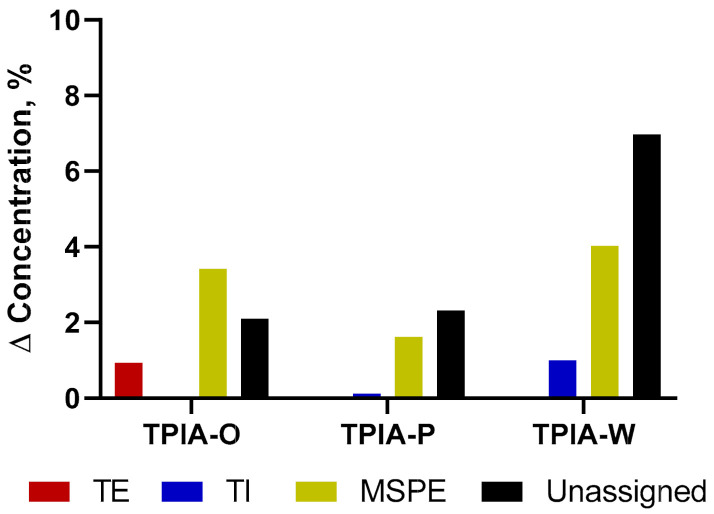
Treprostinil palmitil (TP) and DSPE-PEG2000 degradation products over three months of storage under accelerated conditions (40 °C). TE = Treprostinil Ethyl, TI = Treprostinil Isopropyl, MSPE = 2-stearoyl-sn-glycero-3-phosphoethanolamine-N-[amino(polyethylene glycol)-2000], Unassigned = Sum of all unidentified chromatogram peaks. Note that formation of TE is much greater for treprostinil palmitil inhalation aerosol (TPIA)-O than formation of TI is for TPIA-P.

In the development process, we found that the solubility benefits from increasing amounts of ethanol cosolvent came at the cost of diminished chemical stability. Specifically, we observed transesterification of TP to treprostinil ethyl (TE), an ethyl ester derivative (Supplementary Figure S1). We hypothesized that the use of a more sterically hindered alcohol, such as the secondary alcohol IPA, would result in a diminished transesterification of TP and improved chemical stability (Supplementary Figure S2). Indeed, switching from an EtOH as the cosolvent to IPA resulted in enhanced chemical stability, as demonstrated by the accelerated stability results shown in Figure 2; a more detailed description of the data is available in Table S2 of the supplementary materials supplementary materials. Specifically, during accelerated stability studies, the use of IPA (TPIA-P) resulted in ~8x less TP transesterification degradation relative to the use of EtOH (TPIA-O) after three months of storage at 40 °C. We also observed reduced degradation of the DSPE-PEG2000 excipient when using an IPA cosolvent (Supplementary Figure S3). For each formulation tested, we also observed some materials that were not identified as part of the development process; it is possible that these unidentified compounds represent extractables/leachables, other hydrolysis byproducts (i.e., palmitic acid), or products resulting from PEG400-mediated transesterification of TP, DSPE-PEG2000, or MSPE-PEG2000. Note that for TPIA-W, we observe higher rates of TP and DSPE-PEG2000 degradation than TPIA-P due to the higher concentration of alcohol cosolvent used to promote solubility. As indicated in Table 1, formulation TPIA-X was prepared to mimic 10 mol% degradation of DSPE-PEG200 and evaluate the effect of excipient degradation. Based on the initial solubility and chemical stability screening data, TPIA-W was further evaluated using in vitro (aerosol performance and dose through use) and in vivo studies.

### 2.2. Aerosol Performance

The selection of excipients and relative concentrations of all components can impact MDI aerosol performance [21]. The boiling point and vapor pressure of isopropanol are higher than those of ethanol, which reduces the extent of evaporation that can take place during inhalation [22,23]. As such, we observed diminished aerosol performance for the IPA formulations. To overcome this challenge, we also evaluated different actuator configurations—specifically, actuators with smaller orifice internal diameters and shorter jet lengths [21].

**Figure 3:**
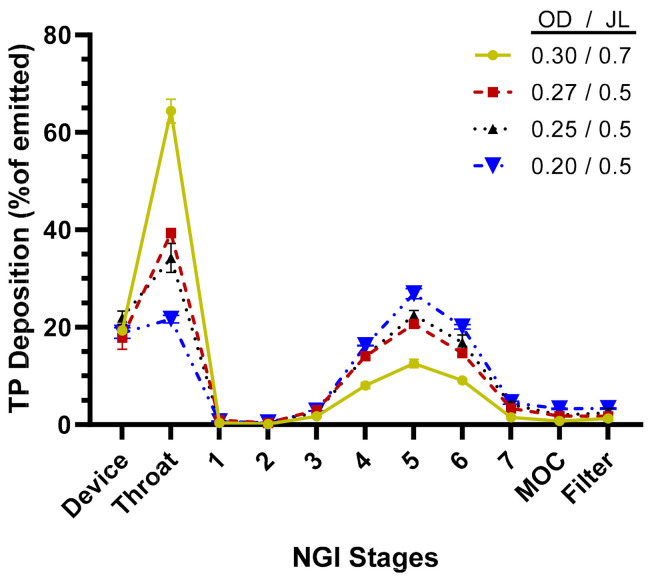
TP distribution across next-generation impactor (NGI) stages for TPIA-W to evaluate the effect of different actuator configurations; OD = actuator orifice diameter (mm), JL = actuator jet length (mm).

The aerosol performance of the TPIA-W formulation was evaluated by conducting aerodynamic particle size distribution (APSD) studies using a next-generation impactor (NGI) at a 30 L/min flow rate and a variety of commercially available actuator devices. The results presented in Table 2 and Figure 3 show that actuator configuration has a significant impact on fine particle fraction (FPF) and fine particle dose (FPD), with smaller actuator size (orifice diameter, OD) leading to increased FPD, reaching to 74% of the emitted dose using a 0.2 mm actuator. Based on the NGI stage deposition data (Figure 3), the effect of reduced actuator size was visible mostly in reduced throat deposition and increased deposition on Stages 4, 5, and 6, corresponding to cutoff aerosol sizes of 2.30, 1.36, and 0.83 µm, respectively.

### 2.3. Dose Through Use

**Figure 4:**
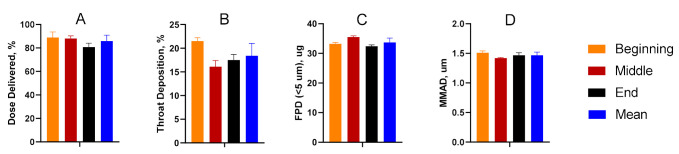
Summary of the delivered dose (**A**), throat deposition (**B**), fine particle dose (FPD) (**C**), and median mass aerodynamic diameter (MMAD) (**D**) throughout the life of the can studies, indicating consistent aerosol performance over time.

Dose-through-use aerosol studies were conducted to evaluate valve performance and assess aerosol stability over time. From the first to final actuation, there was no statistical difference amongst any of the parameters considered. The data suggest that aerosol performance remains unchanged for the TPIA-W formulation irrespective of the number of actuations that had previously occurred (Figure 4). A more detailed presentation of these results is available in the Supplementary Materials (Supplementary Table S3).

### 2.4. Pharmacokinetic Studies

Pharmacokinetic studies were conducted in Sprague Dawley rats using a variety of TPIA formulations to assess the effect of excipients on the TP PK profile. We hypothesized that different excipients, or excipient ratios, would alter the deposition pattern and dissolution kinetics of TPIA and, therefore, change the pharmacokinetic profile of TP. The delivered dose (i.e., the dose presented at the nose of rats) was calculated for each test article in accordance with Equation (1) shown in the Methods Section. It is generally assumed that 10% of the delivered dose is deposited in the lungs. The actual pulmonary dose was determined for each test article based on the concentration of TP in the lungs, the lung weight, and the body weight in accordance with Equation (2). Dose calculations are summarized in Table 3. 

Each of the TPIA formulations tested demonstrated a first-order exponential decay in lung TP and TRE over 24 h with elimination kinetics comparable to those seen with nebulized TPIS. The sum of the lung contents of TP and TRE (originated from TP) was expressed as molar TP equivalent (TP_eq_). These results are illustrated in Table 3. The lung TP_eq_, C_max_, and AU_C0-24h_ values were highest for TPIA-W and lowest for TPIA-S, which is consistent with the varied delivered dose quantified from the filter data. The lung TP_eq_ and T_max_ were identical for each of the TPIA formulations tested (data not shown). The calculated plasma T½ for these formulations varied from a minimum of 2.5 h for TPIA-K to a maximum of 8.5 h for TPIA-W. The plasma T_max_ was very consistent at 0.5 h for all formulations except TPIA-E and TPIA-J, which had T_max_ values of 2.0 h (data not shown). These results indicate that the formulation composition and total delivered dose impact the pharmacokinetic profile.

**Figure 5:**
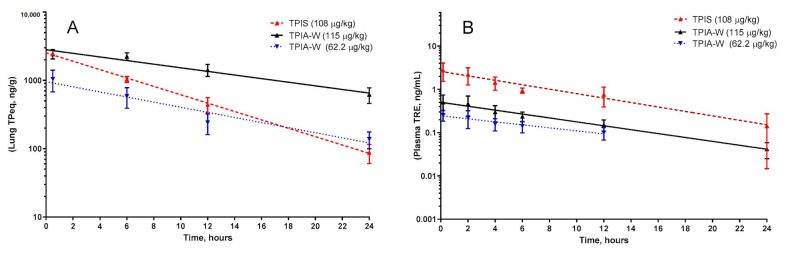
Pharmacokinetic profile of TPIA-W and treprostinil palmitil inhalation suspension (TPIS) showing the lung TP_eq_ PK (**A**) and plasma TRE (**B**). TP_eq_ is the sum of the lung contents of TP and TRE expressed as molar TP equivalent.

For the lead formulation, TPIA-W, lung TP_eq_ had a calculated half-life of 8.01 h when dosed at 62.2 µg/kg and 11.29 h when dosed at 115 µg/kg. A similar trend where increased delivered dose resulted in increased half-life was observed for plasma TRE as well. For TPIA-W, the plasma TRE half-life was 8.5 h when dosed at 62.2 µg/kg and 7.1 h when dosed at 115 µg/kg (Table 3, Figure 5).

As previously mentioned, to evaluate the effect of DSPE-PEG2000 degradation, we prepared formulation TPIA-X, which mimicked TPIA-W in every aspect, except that 10 mol% of DSPE-PEG2000 was replaced by an equal molar amount of MSPE-PEG2000 and palmitic acid in order to mimic a degraded sample. Formulation TPIA-X had intermediate lung TP_eq_ PK parameters and reduced levels of plasma TRE relative to TPIA-W (see Table 3, Supplementary Figure S4).

### 2.5. Cough Studies

**Figure 6:**
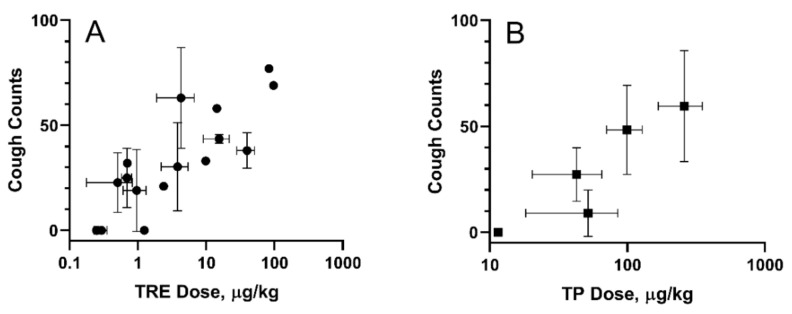
Cough in guinea pigs induced by exposure to inhaled TRE-MDI (**A**) and TPIA (**B**). Error bars represent standard deviation; each point represents data collected from a single experiment day.

A guinea pig cough model was used to evaluate cough response following aerosolized administration of TPIA-W and a positive control, a treprostinil MDI formulation (TRE-MDI), at various doses. Exposure to TRE-MDI caused significant cough at typical delivered doses used in PK studies (Figure 6). To identify the highest delivered dose that did not produce a cough response, i.e., the no-cough dose, we attempted to titrate the dose down until no cough was observed. Based on our data, the highest delivered dose of TRE that did not cause cough in guinea pigs was 0.3 µg/kg. These data confirm that guinea pigs are extremely sensitive to exposure of TRE delivered by MDI [24].

In contrast, exposure of guinea pigs to TPIA-W caused cough response at higher doses than TRE. We observed 48.3 ± 21.1 coughs for a TP pulmonary dose of 73.1 ± 21.5 µg/kg body weight. As the dose was progressively lowered, we observed reduced incidence of cough. A dose–response of cough counts vs. TPIA delivered dose is shown below in Figure 6B. The estimated “no-cough” threshold TP dose was 11.5 ± 0.7 µg/kg (TRE equivalent 8.3 ± 0.5 µg/kg). Exposure of vehicle controls containing either IPA alone or IPA and excipients (DSPE-PEG2000 and PEG400) was well tolerated and did not produce any cough response in guinea pigs (data not shown). We also observed a significant increase in the Pehn index after exposure to MDI-TRE, but not TPIA. When the delivered dose of MDI-TRE was 97 µg/kg, the Penh index increased to almost 900%. 

### 2.6. Efficacy in a Rat Hypoxia Model

**Figure 7:**
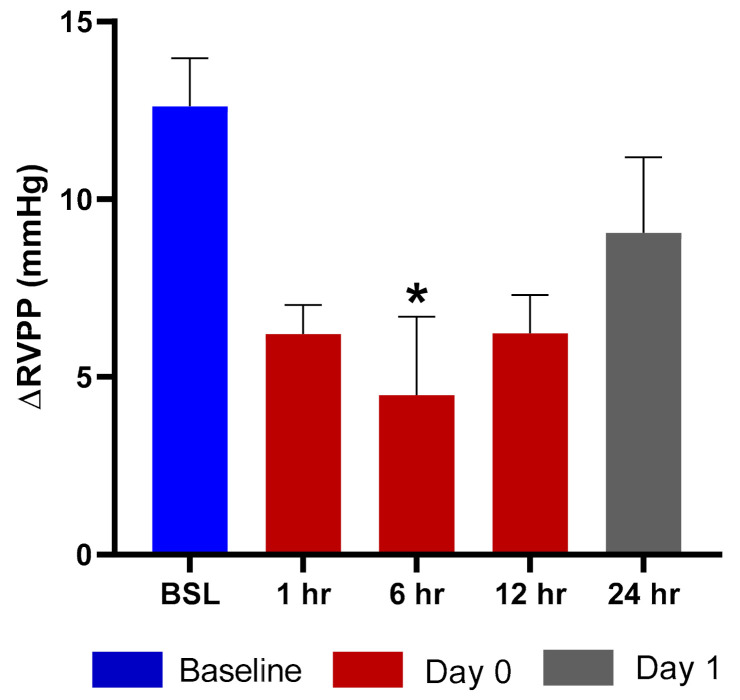
ΔRVPP (right ventricular pulse pressure) response to hypoxic challenge after administration of TPIA-W. * *p* < 0.05 compared to baseline (BSL).

Exposure of rats to hypoxic air causes a transient increase in right ventricular pulse pressure (RVPP) by approximately 10–20 mmHg over the normoxic values [16]. After returning to the normal ambient air, the RVPP is restored to the pre-hypoxia values within 10 min. We evaluated the effects of TPIA by comparing the ∆RVPP (∆RVPP = RVPP Hypoxia − RVPP Normoxia) at various times up to 24 h after drug exposure to the value obtained before the exposure. Exposure to TPIA-W at a delivered dose of 115 µg/kg reduced the hypoxia-induced ∆RVPP, with a maximum reduction of 8.12 mmHg observed at 6 h (Figure 7). The inhibition effect at 12 h was as strong as at 1 h and was still present at 24 h. There was no change to systemic arterial pressure following administration of TPIA.

## 3. Discussion

### 3.1. Formulation

In selecting an appropriate formulation, our goal was to ensure complete dissolution at room-temperature storage and when subjected to cooling–heating cycles. Since TP has limited solubility in pure commercially available propellants (HFA-134a, HFA-227ea, or HP-152a), the use of an alcohol cosolvent was required. The exact amount of alcohol cosolvent needed to ensure a stable solution state in an MDI formulation varied based on the presence of other excipients. We used two types of non-solvent excipients: Excipient 1 was a non-ionic surfactant (pegylated lipid), and Excipient 2 was a hydrophilic glycol (typically PEG400 or PG). The excipients present in our formulation development efforts have been tested previously as excipients in inhaled formulations in animals and humans [25,26,27,28,29,30]. Moreover, similar polyethylene glycol, PEG1000, is used in the approved MDI-based aerosol product CYMBICORT, while DSPE-PEG2000 is a part of injectable liposomal suspension DOXIL and included in the FDA list of “Generally Recognized as Safe” compounds. While this suggests the acceptable safety profile of the chosen excipients, additional toxicology studies of the TPIA may be needed. Both ethanol and IPA are class 3 solvents, according to FDA Guidance Q3C, which states that amounts of these residual solvents of 50 mg per day or less would be acceptable without justification. The amount of IPA in all formulations tested did not exceed 7 mg per actuation, and the daily IPA dose is not expected to exceed the 50 mg limit.

In our formulation, the non-ionic surfactant DSPE-PEG2000 serves as a dispersant after deposition of TPIA aerosol particles in the lungs and helps achieve the target PK profile. The hydrophilic glycol, PEG400, is used as a bulking agent to tune the aerosol size and facilitate dissolution of the TP. It is well established that differences in aerosol particle size distribution are associated with differing deposition profiles within the respiratory tract. Incorporation of PEG400 allows for generation of an aerosol with a relatively consistent droplet size in the respirable range, leading to consistently high deposition in the airways when varying TP and surfactant content. The alcohol cosolvent, isopropyl alcohol present at 10 weight percent, is required to find a balance between formulation solubility and chemical stability. In this application, isopropyl alcohol is preferred over EtOH because its use results in improved chemical stability; the secondary alcohol of IPA is sterically hindered and is therefore less likely to undergo transesterification reactions with TP. Finally, the HFA-134a propellant has a higher vapor pressure than the other tested propellants, which results in improved aerosol performance.

### 3.2. Solubility

Qualitative solubility studies were used to screen formulations using a visual observation technique. In some instances, insoluble material was observed on the walls of the can, either near the liquid–gas interface or on the bottom, or as floating white particulates. Precipitation at the liquid–gas interface may have been caused by high local concentrations of the drug at the interface caused by evaporation and subsequent condensation of the vapor. In some instances, precipitation along the surface of the glass may occur if there is enough surface roughness to cause nucleation; this becomes more likely as the concentration approaches the saturation limit. In other instances, the insoluble material presents as a voluminous floating precipitate that may not be attributed to liquid–gas interface dynamics or surface roughness, and could be attributed to a lack of solubility of the component or components in the formulation. Observation of a clear sample does not necessarily mean that all formulation components are in a homogeneous solution and not a suspension of nanosized particulates, such as micelles. However, it can be used as an effective screening technique to identify viable formulations. Analytical measurement of a formulation in MDI canisters is inherently complicated by the fact that a solvent exists as a liquid in a pressurized canister. Furthermore, the actual solubility state is not critical for the purpose of MDI product, as long as it delivers a consistent drug dose and consistent aerosol properties.

The solubility profile of a specific formulation was dependent on numerous factors, including the relative concentration of each of the formulation components. Most formulations presented as clear homogeneous solutions after manufacturing and during short-term storage at ambient temperature, but failed to remain soluble (i.e., displayed poor physical stability) during longer-term storage (>1 week). These results indicate a kinetic solubility that allowed some in vivo studies to be conducted prior to encountering stability issues. When evaluated at an adequately low temperature, typically –20 °C, we observed formation of a heterogenous suspension for each formulation. To compensate for this observation, our focus shifted towards ensuring re-dissolution of precipitated materials after a short thermal equilibration period. We were encouraged to see that TPIA-W completely re-dissolves after a short room-temperature equilibration period (Table S1).

### 3.3. Aerosol Performance

Aerosol performance of the lead formulation, TPIA-W, was optimized by evaluating the effect of actuator geometry on different aerosolization parameters using four different MDI actuators. For solution-based MDI formulations, the container closure system plays an integral role in the generation of the aerosol cloud. The major factors that influence delivery efficiency are the HFA–cosolvent ratio, actuator orifice diameter, metering valve volume, and the net concentration of non-volatile components. The container closure system of a solution MDI system consists of the canister, metering valve, and actuator. In general, lower concentrations of cosolvents and non-volatile components, as well as smaller orifice diameters and metering volumes, are associated with the generation of finer aerosol clouds, which result in more efficient atomization. Indeed, the results presented in Table 2 and Figure 3 confirm that actuator configuration has a significant impact on aerosol performance, specifically FPF, with smaller actuator size (orifice diameter) leading to increased FPF, reaching to 74% of the emitted dose using a 0.2 mm actuator. The effect of actuator size was most noticeable in terms of reduced throat deposition and increased deposition on Stages 4, 5, and 6 of an NGI, corresponding to cutoff aerosol sizes of 2.30, 1.36, and 0.83 µm, respectively (Figure 3).

These results highlight the intricate balance between solubility performance, aerosol performance, and patient convenience. Too high of a drug loading and the solubility suffers, while too much cosolvent and the aerosol performance suffers. In theory, both challenges can be solved by reducing the drug loading; however, if the fine particle dose drops below the therapeutic dose, multiple actuations may be required per administration event, which could reduce patient convenience and, potentially, patient compliance.

### 3.4. Pharmacokinetics

Building on our previous experience with TRE prodrugs, we anticipated the efficacy response of our drug to be based predominantly on the concentration of TRE, the active ingredient, in the lungs, rather than the plasma concentration of TRE [17]. As such, criteria for our target PK profile included (1) slow clearance of TP from the lungs to sustain efficacious lung TRE levels up to 24 h, (2) negligible progressive accumulation of TP in the lungs, and (3) plasma TRE C_max_ values significantly lower than TRE delivered by MDI. Low concentration of TRE in the plasma reduces potential adverse systemic events, such as reductions in systemic blood pressure.

Most TPIA formulations exhibited a similar PK profile to nebulized TPIS with the highest levels of TP and TRE being found in the lungs immediately after dosing (0.5 h), with an exponential decay over 24 h. However, there were some notable exceptions to this trend. For example, there was significant TP remaining in the lungs 24 h after administration for TPIA-A, and lung T_1/2_ for TP_eq_ was 15 h, which was prepared without any excipients. This would present a potential concern because repeating once-daily administration may result in drug accumulation, which does not align with our PK criteria. 

Based on a compromise between chemical stability (IPA), physical stability (10% w/w alcohol cosolvent), and in vivo performance (PK, efficacy, and cough), TPIA-W was selected as a lead formulation. When compared directly to a nebulized TPIS formulation, the TP_eq_ PK profile of the lead TPIA formulation, TPIA-W, is comparable for lung and plasma measurements, as shown in Figure 5 [31]. Interestingly, there does appear to be a significant difference in the lung TP_eq_–plasma TRE C_max_ ratio for the different formulations. The lung/plasma C_max_ ratio for nebulized TPIS is around 800, while the same ratio for TPIA-W is around 4100–4800 and appears to be dependent on the dose. This observation is significant because it suggests that inhaled formulations delivered by MDI may have lower systemic exposure than nebulized formulations.

### 3.5. Cough

A common adverse event associated with inhaled TRE in humans is cough [1]. In guinea pig models, we have shown that TRE-induced cough is mediated by activation of prostanoid IP receptors in the airways [27]. Building on our previous work, we decided to use guinea pigs as a model to study the effect of aerosol administration of TP and TRE delivered by MDI on cough and ventilation. The cough response provoked by nebulized TRE occurs at a substantially lower inhaled dose compared to TP delivered by nebulization or by dry powder [16,27]. In addition, significant increase of the Penh index at the delivered dose of MDI-TRE of 97 µg/kg was indicative of a breathing pattern typically observed during bronchoconstriction [32,33]. We evaluated progressively lower doses of TRE to identify the highest dose that did not cause significant impact on cough and ventilation. This was a significant challenge, as we had to significantly reduce the formulation strength, actuation frequency, and dose duration for MDI-TRE relative to TPIA. Ultimately, we identified a TRE delivered dose of 0.26 ± 0.03 µg/kg body weight as the highest dose that would not produce a cough response. This result is significant because it confirms that only a small amount of TRE delivered by an MDI is required to elicit a cough response. These results mandate that we control formulation stability and limit transesterification and hydrolysis reactions that could lead to appreciable formation of TRE. Furthermore, these results suggest that cough is a critical parameter for assessing new drug formulations, as conversion to TRE would lead to significant side effects.

For all the experiments, the measured delivered dose of TRE or TP correlated with the numbers and frequency of the actuations. In addition, the MDI vehicles did not induce cough or changes in respiratory parameters. A dose–response was observed for studies done with TPIA, and the data are presented in Figure 6. As the dose was progressively lowered, we observed reduced incidence of cough. For the lowest TP delivered dose considered in this experiment of 11.5 µg/kg (TRE equivalent 8.3 ± 0.6 µg/kg) body weight, we observed zero cough response. This “no cough“ dose represents a 32-fold increase relative to that for MDI-TRE. Analysis of different respiratory parameters, including tidal volume, respiratory rate, and minute volume, demonstrated that no significant changes were observed, suggesting that these delivered doses would not cause bronchoconstriction.

### 3.6. Efficacy

Efficacy was assessed using rats that were prepared with telemetry probes implanted in the right ventricle and descending aorta to measure the increase in RVPP and the change in and mean systemic arterial blood pressure (mSAP) induced by exposure to acute hypoxia. This model had previously been used to evaluate nebulized TP formulations that demonstrated potent and long-acting inhibition of the hypoxia-induced increase in RVPP with no change in mSAP [34]. Exposure to TPIA at a delivered dose of 115 µg/kg strongly reduced the hypoxia-induced ∆RVPP response for at least 12 h, with the maximum and statistically significant (*p* < 0.05) inhibition observed at 6 h (Figure 7) and some RVPP reduction at 24 h. These data suggest that a single dose of TPIA provides sustained efficacy, though a higher delivered dose may be required to achieve once-daily administration. This would have to be evaluated and confirmed in clinical trials as part of a clinical development program.

## 4. Materials and Methods

### 4.1. MDI Canisters

MDI canisters were prepared using a two-stage filling method. Briefly, an appropriate amount of concentrated feedstock solution containing the active drug and all excipients was added to an empty canister. Canisters were made of aluminum, PVC coated glass, or PET depending on the experiment. A valve was then crimped in place and propellant was added using a Pamasol Lab-2016 manual filling station. The crimp height was set based on valve manufacturer specs (5.71 mm for the Aptar DF 316/50 RCU CS20 ARGENT valve) and was measured using a Socoge International Crimper-Control (model Ø20:743-03-143). Mass measurements were made throughout the manufacturing process to calculate formulation compositions. The exact formulation compositions were measured using dose uniformity and total can content assays.

### 4.2. Solubility

Qualitative solubility studies were used to screen different formulations for solution stability. Samples were prepared in PVC-coated glass or PET canisters and were subjected to a visual examination looking for precipitation of insoluble material. Typically, insoluble material was observed on the walls of the can, either near the liquid–gas interface or on the bottom, or as floating white particulates.

To conduct the time to precipitate/time to re-dissolve experiment, five glass canisters containing TPIA-W were placed at 5 and 15 °C separately for 24 h. A refrigerator was used to maintain the 5 °C storage conditions. To maintain the 15 °C storage conditions, the lyophilizer was adjusted so that the ambient temperature was 15 °C. After a 24-h incubation period was completed, all canisters containing precipitate were placed on the lab bench under ambient conditions (i.e., room temperature) and were observed visually at 30 min intervals to track the time to re-dissolve.

### 4.3. Aerosol Performance

Aerosol performance was evaluated by characterizing the APSD of TP in the MDI formulation by determination of MMAD, the geometric standard deviation (GSD), the diameter under which 90% of the cumulative distribution falls (D90), the diameter under which 10% of the cumulative distribution falls (D10), the FPF < 5 µm, and the FPD < 5 µm. All APSD testing was performed at a 30 L/min flowrate at environmental conditions of 23°C and 50% relative humidity using an induction port and adapter, pre-separator, next-generation impactor (NGI), and filter. Before each experiment, the MDI canister was primed three times into a waste container and then actuated 10 times for each experiment to ensure that enough TP was deposited for quantitation. Total recovery from APSD testing was determined from the emitted dose for a combined 10 actuations via a delivered dose uniformity (DDU) method using a dose uniformity sampling apparatus (DUSA). Quantitation of TP deposited on each NGI stage was accomplished via a calculation of an external seven-point linear log-log calibration curve of the log of the peak area of TP versus the log of the standard concentration over the nominal range of 0.4 to 25 µg/mL using high-performance liquid chromatography (HPLC) and a charged aerosol detector (CAD) on a C8 column.

### 4.4. Dose through Use

Dose through use was evaluated by performing DDU and APSD throughout the life of canisters during storage under ambient conditions. APSD characterization and DDU testing were conducted at the first actuation (after priming; beginning), 100th actuation (middle), and 200th actuation (end), while on every other non-APSD characterization day, the cans were actuated 10 times to measure the emitted mass by the gravimetric method for each actuation. Canisters were actuated using an H&T PressPart actuator with a 0.2 mm orifice internal diameter and a 0.5 mm jet length. DDU testing was performed using a DUSA for a combined two actuations at 28.3 L/min vacuum flowrate. Quantitation of the emitted TP dose was accomplished using the same HPLC-CAD method described above for quantitation of TP deposited on each NGI stage.

### 4.5. Chemical Stability

Chemical stability was evaluated by analyzing samples collected using a standard Total Can Content (TCC) assay. For TCC experiments, MDI canisters were chilled, valves were removed (InnovaSystems MDI AC2), and volatile contents were allowed to gradually warm to room temperature, allowing for gentle evaporation of the propellant. The remaining solution was diluted for quantitation using the HPLC-CAD method described above.

### 4.6. Inhalation Studies

Inhalation studies in rats were performed using a nose-only inhalation tower modified for MDI delivery. For each study, the weight of the canisters was measured before and after the study, and the duration of actuation was recorded. On the day of the study, the rats were placed in restraining tubes that were connected to the exposure ports of the inhalation tower, comprising an automated actuator (for a maximum of six canisters), three levels of exposure ports with each level having 20 exposure ports for the rats, and a base unit (Supplementary Figure S5). MDI formulations were actuated into the tower with tangential airflow of 2 L/min and a vacuum pull of 20 L/min at the base unit. Cohorts of 11 rats were used with a filter connected to one open exposure port, from which the aerosol concentration of TP was measured. A vacuum pump was connected to the filter and set at a vacuum flow of either 2.0 or 3.0 L/min, which began at 5 min after the start of the aerosolization of the drug and ended 1, 2, or 3 min later; i.e., a filter sampling time of 1, 2, or 3 min depending on the formulation concentration and the number of canisters used. The filter samples were analyzed for TP measured by HPLC with a CAD detector.

Following exposure to the drugs, blood and lung tissue samples were obtained from each rat at times of 0.5, 2, 4, 6, 12, and 24 h for blood and 0.5, 6, 12, and 24 h for lungs. The 0.5 h time point was defined as the immediate post-dose (IPD) sampling time. The plasma was separated from the blood samples and the lung tissues were homogenized to measure the concentrations of TRE and TP by HPLC MS/MS. The concentrations of TP and TRE in the lungs were expressed as either their absolute values or combined into a single value and expressed as the TP equivalent value (TP_eq_). The conversion of TRE into molar equivalents of TP involved multiplication of the TRE concentration by a factor of 1.575 and is based upon the molecular weights of TP and TRE, which are 614.9 and 390.5 g/mol, respectively.

### 4.7. Pharmacokinetic Analysis

Analysis of the plasma and lung pharmacokinetics was performed with PKSolver program, which is an add-in program for Microsoft Excel [35]. The “Non-Compartmental Analysis after Extravascular Input” module was used to calculate PK parameters.

### 4.8. Dose Calculations

Delivered (at the nose) dose was calculated for each test article based on the aerosol filter TP concentration (CF), respiratory minute volume (RMV), exposure duration of the rats (D), deposition fraction (DF) of 100%, and body weight (BW) of the rats in accordance with Equation (1), which is shown below [36]. The RMV was calculated using the formula: RMV (L/min) = 0.608 x BW (kg) 0.852 [36]. The pulmonary dose was calculated for each test article based on the concentration of TP in the lungs (CL) immediately post-dose (0.5 h), the lung weight (LW), and the body weight (BW), in accordance with Equation (2), which is shown below [36].

(1) $$Delivered\;Dose\;\left(\frac{µg}{kg}\right)=\;\frac{CF\;\left(\frac{µg}{L}\right)x\;RMV\;\left(\frac{L}{min}\right)x\;D\;\left(min\right)x\;DF\;}{BW\;\left(kg\right)}$$

(2) $$Pulmonary\;Dose\;\left(\frac{µg}{kg}\right)=\;\frac{CL\;\left(\frac{µg}{g}\right)x\;LW\;\left(g\right)\;}{BW\;\left(kg\right)\;}$$

### 4.9. Cough Methods

Experiments were performed in male Hartley guinea pigs. After a three-day period of acclimation, the guinea pigs were placed in a whole-body plethysmograph for the measurement of ventilation (tidal volume, respiratory rate, and minute volume), Penh, and cough using established techniques [16,27,32,33]. Cough was measured from plethysmograph recordings showing a large inspiration followed by a large expiration and confirmed by manual observations, video recordings, and cough sounds [16]. Test articles were delivered via MDI for 15 min, followed by a 120-min observation period after the aerosol compounds were given. Ventilation, Penh, and cough were measured before, during, and after exposure to the test articles. At the end of the study, plasma samples and respiratory tissues of lungs were collected. All plasma and respiratory tissue samples were analyzed for their concentration(s) of TP and/or TRE.

### 4.10. Efficacy and PK Determinations

Experiments were performed in male Sprague Dawley rats that were implanted with telemetry probes in the right ventricle and descending aorta to measure RVPP and mean systemic arterial blood pressure (mSAP) for efficacy studies and in rats with no surgical intervention for the PK studies. In telemetered rats, the cardiovascular parameters were measured at specified intervals during the hypoxia challenge experiment, including a 10-min interval while breathing normoxic air (21% O_2_/balance N_2_) and a 10-min interval immediately after exposure to hypoxic air (10% O_2_/balance N_2_). The increase in RVPP due to the hypoxia challenge (ΔRVPP due to hypoxia) was measured before drug exposure and at 1, 6, 12, and 24 h after exposure to inhaled test articles, as previously described [34]. Nose-only inhalation studies were performed in the same manner as the PK studies described above. In the PK rats, plasma samples were collected at times of 0.5, 2, 4, 6, 12, and 24 h, and respiratory tissues of lungs were collected at times of 0.5, 6, 12, and 24 h after drug exposure. All plasma and respiratory tissue samples were analyzed for their concentration(s) of TP and/or TRE.

## 5. Conclusions

A series of metered-dose inhaler formulations of TP, called TPIA, have been developed for potential treatment of pulmonary arterial hypertension. Using an iterative evaluation process focused on in vitro performance parameters, such as chemical stability, physical stability, and aerosol performance, we were able to identify a lead formulation, TPIA-W, which consists of TP, DSPE-PEG2000, and PEG400 at a concentration ratio of 1:0.5:3.0 mg/mL, dissolved in HFA-134a/IPA cosolvent-based propellant. DSPE-PEG2000 and PEG400 are both novel MDI excipients. DSPE-PEG2000 is a non-ionic surfactant that serves as a dispersant after deposition of TPIA aerosol particles in the lungs and helps achieve the target PK profile. PEG400 is a hydrophilic glycol that is used as a bulking agent to tune the aerosol size and facilitate dissolution of the TP. Combining this formulation with an optimized device, an actuator equipped with a 0.2 mm orifice internal diameter and 0.5 mm jet length, we were able to achieve an MMAD of 1.54 µm and an FPF of 0.74.

Confirmatory in vivo testing demonstrated an acceptable PK profile for once-daily inhalation, efficacy for at least 12 h, and a reduced cough response relative to inhaled treprostinil. Specifically, the PK profile of the TPIA-W formulation was similar to a previously reported PK profile of the nebulized formulation of TPIS, demonstrating slow clearance of the TP prodrug within 24 h while maintaining reduced TRE systemic exposure (~5000 times lower AUC_0-24h_) as compared to TP_eq_ lung exposure. Additionally, cough studies indicated that the “no-cough” dose of TPIA is approximately 32-fold higher than that of TRE-MDI aerosol, providing additional evidence that masking the carboxylic acid of TRE using an ester prodrug strategy can result in decreased cough response following inhaled administration [16]. Finally, efficacy studies involving hypoxic challenges in telemetered rats confirmed the vasodilatory effects of single-dose TPIA for at least 12 h. Overall, the TPIA formulation appears to demonstrate a promising preclinical profile that warrants further evaluation for its development into a clinical candidate.

## Figures and Tables

**Table 1:** Summary of the targeted compositions for selected metered-dose inhaler (MDI) formulations.

TPIA	Excipient 1	Excipient 2	Alcohol	Propellant	Composition (mg/mL)			Alcohol (wt %)
					TP	Exc1	Exc2	
TPIA-A	DSPE-PEG2000	-	EtOH	HFA-134	1	0.5	0	9
TPIA-C	DSPE-PEG2000	PEG400	EtOH	HFA-227	0.5	0.25	0.75	5
TPIA-E	DSPE-PEG2000	-	EtOH	HFA-227	3	3	0	10
TPIA-H	DSPE-PEG2000	PG	EtOH	HFA-227	1	0.5	1.5	3
TPIA-I	DSPE-PEG2000	PEG1000	EtOH	HFA-227	1	0.5	3	3
TPIA-J	DSG-PEG2000	PEG400	EtOH	HFA-227	0.5	0.25	3	5
TPIA-K	DPG-PEG2000	PEG400	EtOH	HFA-227	0.5	0.25	3	5
TPIA-L	DSPE-PEG2000	PEG400	EtOH	HFA-227	0.5	0.25	3	5
TPIA-O	DSPE-PEG2000	PEG400	EtOH	HFA-227	1	0.5	6	7
TPIA-P	DSPE-PEG2000	PEG400	IPA	HFA-227	1	0.5	6	7
TPIA-S	Brij-58	PEG400	EtOH	HFA-227	1	0.5	6	5
TPAI-T	Brij-58	PEG400	IPA	HFA-134	1	0.5	3	7
TPIA-W	DSPE-PEG2000	PEG400	IPA	HFA-134	1	0.5	3	10
TPAI-X	DSPE-PEG2000 ^1^	PEG400	IPA	HFA-134	1	0.5	3	10

^1^ Formulation X was prepared with 10 mol% DSPE-PEG2000 substituted by an equal amount of MSPE-PEG2000 and palmitic acid to mimic a degraded sample.

**Table 2:** Summary of aerodynamic particle size distribution (APSD) measurements for evaluating the effect of actuator configuration on TPIA-W aerosol performance.

Jet Length (mm)	Orifice Diameter (mm)	MMAD (µm)		Fine Particle Fraction (%)		Fine Particle Fraction (%)	
		Mean	SD	Mean	SD	Mean	SD
0.30	0.7	1.69	0.04	33.5	2.3	14.7	0.69
0.27	0.5	1.72	0.04	56.1	0.9	25.7	1.48
0.25	0.5	1.61	0.1	61.8	3.6	27.5	0.41
0.2	0.5	1.54	0.03	74.2	0.9	34.9	1.28

**Table 3:** Summary of the delivered dose, lung TP_eq_, and plasma TRE PK following nose-only aerosol administration of TPIA formulations in rats. NC = Not calculated. TP_eq_ is the sum of the tissue content of TP and TRE expressed as molar TP equivalent.

Test Article	TP Delivered Dose (µg/kg)	Lung TP_eq_ C_max_ (ng/g)	Lung TP_eq_ AUC_0-24_ (µg·h/g)	Lung TP_eq_ T_1/2_ (h)	Plasma C_max_ (ng/mL)	Plasma TRE AUC_0-24_ (ng·h/mL)	Plasma TRE T_1/2_ (h)
TPIS	108	24700	17.9	4.9	2.79	20.97	5.8
TPIA-A	10.8	765	9.5	15	NC1	NC1	NC1
TPIA-E	29.9	1430	12.9	9.14	0.14	1.32	7.8
TPIA-H	67.3	3080	36.2	10.2	0.22	2.23	4.7
TPIA-I	66.8	1670	19.4	10.8	0.33	2.54	3.2
TPIA-J	12.1	438	3.5	5.59	0.07	0.28	4.9
TPIA-K	26.4	436	4.2	5.8	0.95	1.46	2.5
TPIA-L	13.2	1190	11.2	6.05	NC	NC	NC
TPIA-S	12.5	237	2.01	4.67	NC	NC	NC
TPIA-W	115	2440	36.8	11.3	0.5	4.53	7.1
	62.2	1040	9.5	8.01	0.25	1.84	8.5
TPIA-X	111	1850	16.9	7.99	0.42	3.6	3.9

## Data Availability

Data is contained within the article and supplementary material.

## Supplementary Materials

The following are available online at https://www.mdpi.com/1422-0067/22/2/548/s1.

## Abbreviations

API	Active Pharmaceutical Ingredient
APSD	Aerodynamic Particle Size Distribution
BSL	Baseline
BW	Body Weight
CAD	Charged Aerosol Detector
CF	Aerosol Concentration of the Filter
CL	Concentration of TP in the Lungs
D	Exposure Duration
DDU	Delivered Dose Uniformity
DF	Deposition Fraction
DSPE-PEG2000	1,2-distearoyl-sn-glycero-3-phosphoethanolamine-N-[amino(polyethylene glycol)-2000]
DUSA	Dose Uniformity Sample Apparatus
EtOH	Ethanol
FPD	Find Particle Dose
FPF	Fine Particle Fraction
GSD	Geometric Standard Deviation
HFAHPLC	HydrofluoroalkaneHigher-Performance Liquid Chromatography
IPA	Isopropyl Alcohol
JL	Jet Length
LW	Lung Weight
MMADMDI	Median Mass Aerodynamic DiameterMetered-Dose Inhaler
MS	Mass spectrometry
mSAP	Mean Systemic Arterial Blood Pressure
MSPE-PEG2000	2-stearoyl-sn-glycero-3-phosphoethanolamine-N-[amino(polyethylene glycol)-2000]
NGI	Next-Generation Impactor
OD	Orifice Diameter
PAH	Pulmonary Arterial Hypertension
PEG	Polyethylene Glycol
PET	Polyethylene Terephthalate
PG	Propylene Glycol
PK	Pharmacokinetics
PVC	Polyvinyl Chloride
RMV	Respiratory Minute Volume
RVPP	Right Ventricular Pulse Pressure
TCC	Total Can Content
TE	Treprostinil Ethyl
TI	Treprostinil Isopropyl
TP	Treprostinil Palmitil
TPeq	Treprostinil Palmitil Equivalents
TPIA	Treprostinil Palmitil Inhalation Aerosol
TRE	Treprostinil
