# Supplementary material for: Development and Characterization of Treprostinil Palmitil Inhalation Aerosol for the Investigational Treatment of Pulmonary Arterial Hypertension
Source: Int J Mol Sci. 2021 Jan 7;22(2):548. doi: 10.3390/ijms22020548 (PMC7828008; doi:10.3390/ijms22020548)
Supplement: Supplementary file 1 [file ijms-22-00548-s001.pdf]

International Journal of
Molecular Sciences

MDPI

Supplemental Material

Development and Characterization of Treprostinil Palmitil Inhalation Aerosol for the Investigational Treatment of Pulmonary Arterial Hypertension

Adam J. Plaunt, Sadikul Islam, Tony Macaluso, Helena Gauani, Thomas Baker, Donald Chun, Veronica Viramontes, Christina Chang, Michel R. Corboz, Richard W. Chapman, Zhili Li, David C. Cipolla, Walter R. Perkins and Vladimir S. Malinin *

Insmed Incorporated, Bridgewater, NJ 08807, USA; adam.plaunt@insmed.com (A.J.P.); Sadikul.Islam@Insmed.com (S.I.); Antonio.Macaluso@Insmed.com (T.M.); Helena.Gauani@insmed.com (H.G.); Tomas.Baker@insmed.com (T.B.); Donald.Chun@insmed.com (D.C.); Veronica.Viranontes@insmed.com (V.V.); Christina.Chang@Insmed.com (C.C.); Michel.Corboz@Insmed.com (M.C.R.); Richard.Chapman@Insmed.com (R.W.C.); Zhili.Li@insmed.com (Z.L.); David.Cipolla@Insmed.com (D.C.C.); Walter.Perkins@insmed.com (W.R.P.)

* Correspondence: vladimir.malinin@insmed.com; Tel.: +1-732-487-7378

Received: 29 November 2020; Accepted: 2 January 2021; Published: 7 January 2021

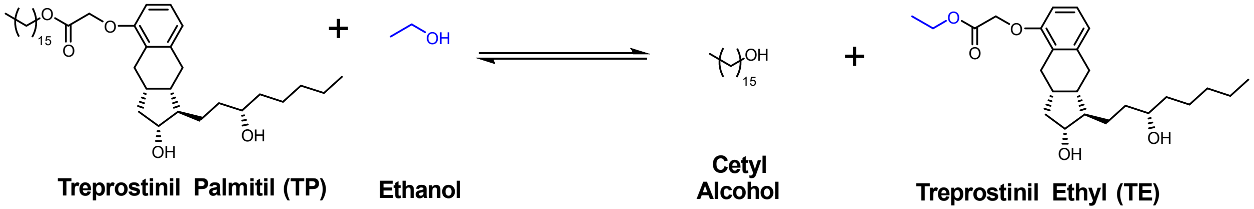

Figure S1. EtOH mediated transesterification of TP and EtOH to TE and Cetyl Alcohol.

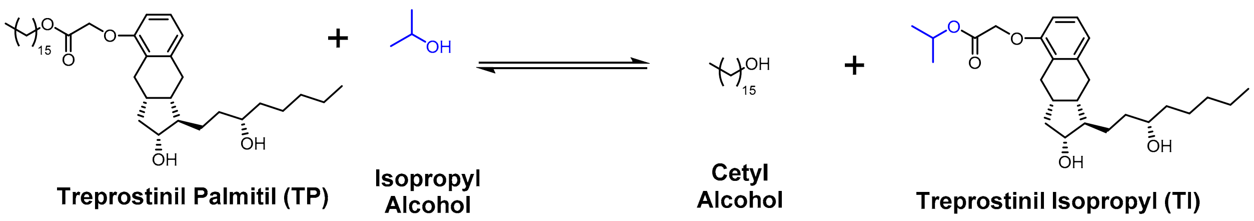

Figure S2. IPA mediated transesterification of TP and IPA to TI and Cetyl Alcohol.

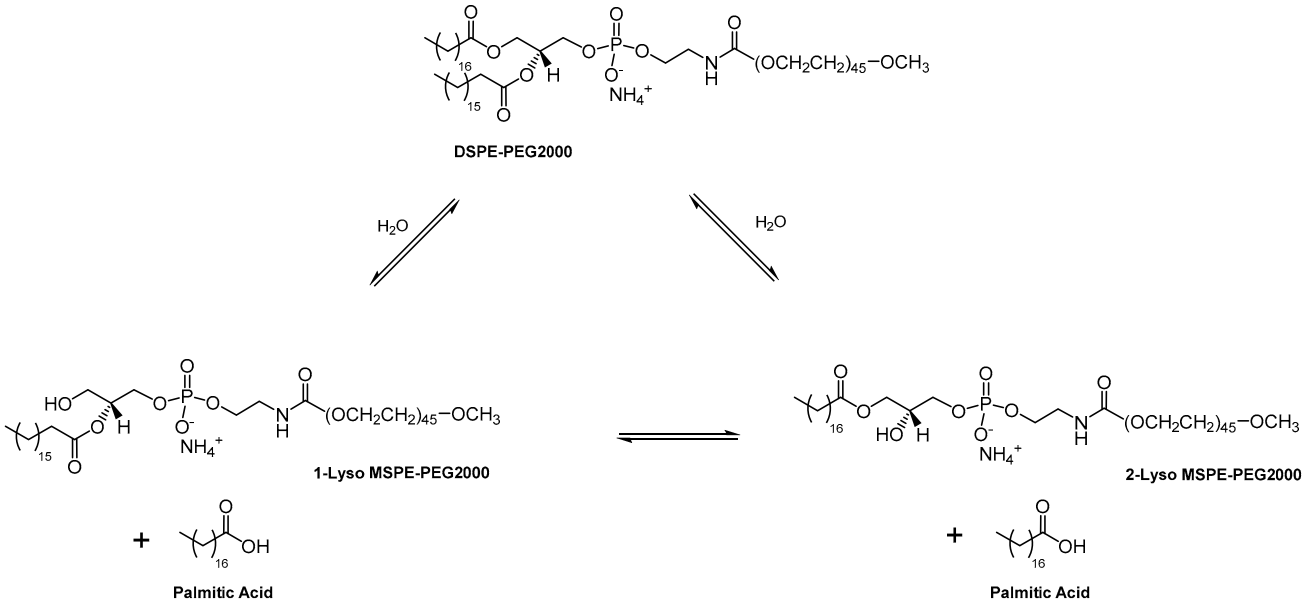

Figure S3. Hydrolytic degradation of DSPE-PEG2000 to MSPE-PEG2000. Note that degradation can form either the 1-Lyso or the 2-Lyso derivatives of MSPE-PEG2000 and that these forms exist in equilibrium.

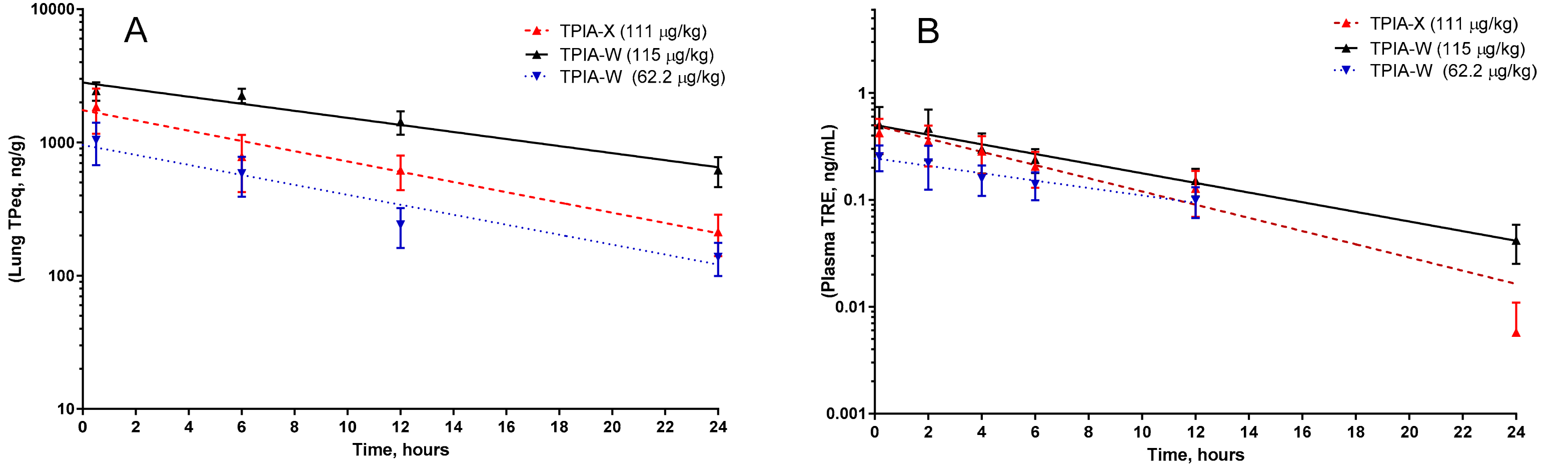

Figure S4. Pharmacokinetic profile of TPIA-X and TPIA-W showing the lung TPeq PK (A) and plasma TRE (B).

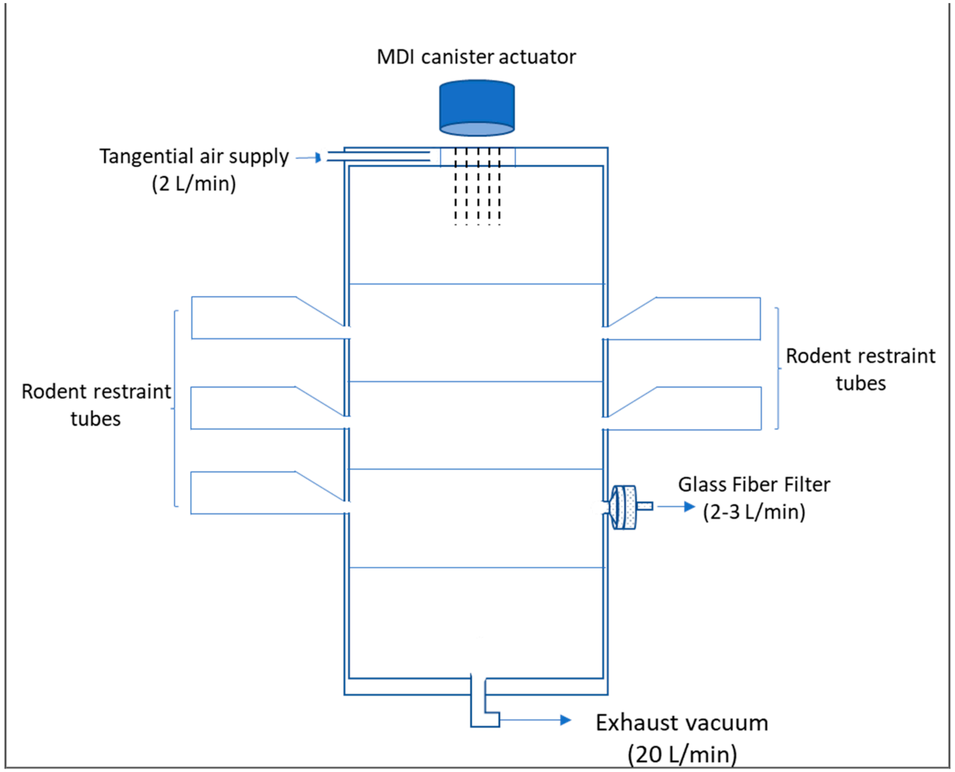

Figure S5. A Schematic Diagram of TPMDI Nose-Only Exposure System.

Table S1. Summary of targeted compositions for selected MDI Formulations.

**Formulation**	**Temp (°C)**	**Solubility Observation**[2]							
		**0 h**	**1 h**	**2 h**	**3 h**	**4 h**	**5 h**	**6 h**	**23 h**
TPAI-W	5	CS	CS	CS	CS	PPT	PPT	PPT	PPT
TPIA-W Control[1]	5	CS	CS	CS	CS	CS	CS	CS	CS
TPIA-W	15	CS	CS	CS	CS	CS	CS	CS	CS
TPIA-W Control[1]	15	CS	CS	CS	CS	CS	CS	CS	CS

[1] * TPIA-W Control = DSPE-PEG2000, and PEG400, at a concentration ratio of 0.5:3.0 mg/mL dissolved in HFA-134a with 10 % IPA (by weight). [2]. CS = Clear Solution, PPT = Precipitate.

Table S2. Summary of APSD measurements evaluating the effect of actuator configuration on TPIA-W aerosol performance.

Formulation	Time	TP		TE		TI		DSPE-PEG2000		MSPE-PEG2000		Other	
		Mean	SD	Mean	SD	Mean	SD	Mean	SD	Mean	SD	Mean	SD
TPIA-O	0M	45.18	0.16	0.00	0.00	0.00	0.00	54.82	0.16	0.00	0.00	0.00	0.00
	1M	44.89	0.17	0.44	0.04	0.00	0.00	52.24	0.12	1.73	0.11	0.69	0.15
	2M	40.62	3.15	0.81	0.05	0.00	0.00	53.50	3.15	3.05	0.11	2.01	0.06
	3M	41.28	3.52	0.94	0.15	0.00	0.00	52.09	4.16	3.42	0.21	2.10	0.28
TPIA-P	0M	45.55	0.14	0.00	0.00	0.00	0.00	54.40	0.23	0.00	0.00	0.06	0.10
	1M	43.41	0.09	0.00	0.00	0.00	0.00	55.75	0.01	0.75	0.01	0.10	0.04
	2M	41.92	0.22	0.00	0.00	0.11	0.02	54.67	0.35	1.34	0.14	1.95	0.49
	3M	42.25	0.83	0.00	0.00	0.12	0.01	53.32	0.70	1.62	0.01	2.38	0.39
TPIA-W	0M	46.47	0.17	0.00	0.00	0.00	0.00	53.41	0.13	0.25	0.00	0.25	0.00
	1M	46.54	0.20	0.00	0.00	0.11	0.00	52.80	0.24	0.53	0.08	0.91	0.09
	2M	41.92	0.22	0.00	0.00	0.73	0.12	54.67	0.35	4.27	0.12	6.79	0.61
	3M	51.80	0.20	0.00	0.00	1.00	0.05	43.77	0.10	4.28	0.12	7.23	0.34

Table S3. Summary table showing results from Dose Through Use experiments for TPIA-W.

Parameter	Dose Delivery Ex-Device, %)		MMAD (µm)		GSD		% Throat Deposition		% FPF (< 5 µm)		FPD (<5 µm), µg		APSD % Mass Balance	
	**Mean**	**SD**	**Mean**	**SD**	**Mean**	**SD**	**Mean**	**SD**	**Mean**	**SD**	**Mean**	**SD**	**Mean**	**SD**
Beginning	88.9	4.7	1.51	0.03	2.02	0.02	21.5	0.7	75.0	0.4	33.26	0.47	88.7	1.1
Middle	88.0	2.3	1.42	0.01	1.98	0.02	16.1	1.3	81.2	1.4	33.51	0.42	87.5	2.6
End	80.7	3.3	1.47	0.04	2.13	0.03	17.5	1.2	78.2	1.6	32.41	0.48	83.0	2.0
Mean	85.8	5.1	1.47	0.05	2.04	0.07	18.4	2.6	78.1	2.9	33.73	1.44	86.4	3.1
